# From Murine to Human Nude/SCID: The Thymus, T-Cell Development and the Missing Link

**DOI:** 10.1155/2012/467101

**Published:** 2012-03-05

**Authors:** Rosa Romano, Loredana Palamaro, Anna Fusco, Leucio Iannace, Stefano Maio, Ilaria Vigliano, Giuliana Giardino, Claudio Pignata

**Affiliations:** Department of Pediatrics, “Federico II” University, Via Pansini 5, 80131 Naples, Italy

## Abstract

Primary immunodeficiencies (PIDs) are disorders of the immune system, which lead to increased susceptibility to infections. T-cell defects, which may affect T-cell development/function, are approximately 11% of reported PIDs. The pathogenic mechanisms are related to molecular alterations not only of genes selectively expressed in hematopoietic cells but also of the stromal component of the thymus that represents the primary lymphoid organ for T-cell differentiation. With this regard, the prototype of athymic disorders due to abnormal stroma is the Nude/SCID syndrome, first described in mice in 1966. In man, the DiGeorge Syndrome (DGS) has long been considered the human prototype of a severe T-cell differentiation defect. More recently, the human equivalent of the murine Nude/SCID has been described, contributing to unravel important issues of the T-cell ontogeny in humans. Both mice and human diseases are due to alterations of the FOXN1, a developmentally regulated transcription factor selectively expressed in skin and thymic epithelia.

## 1. Introduction

Primary immunodeficiencies (PIDs) are severe disorders of the immune system in which patients cannot produce a proper protective immune response, leading to an increased susceptibility to infections. Nowadays, more than 200 well-characterized genetic immune deficiencies have been identified thanks to the advances in molecular genetics and immunology. PIDs are classified according to the component of the immune system that is primarily involved including T, B, natural killer (NK) lymphocytes, phagocytic cells, and complement proteins [1].

Primary T-cell defects are rare disorders, accounting for approximately 11% of reported PIDs [2]. These diseases may be considered true experiments of the nature in that the recognition of the molecular mechanisms underlying their pathogenesis led to clarify the phases of the T-cell differentiation process and the physiological mechanisms of the T-cell responses. Studies in this field led to unravel the checkpoints, which play a pivotal role in these processes, which mostly rely on a proper intercellular interaction between thymocytes and the thymic microenvironment.

## 2. T-Cell Development and Thymus

**Figure 1:**
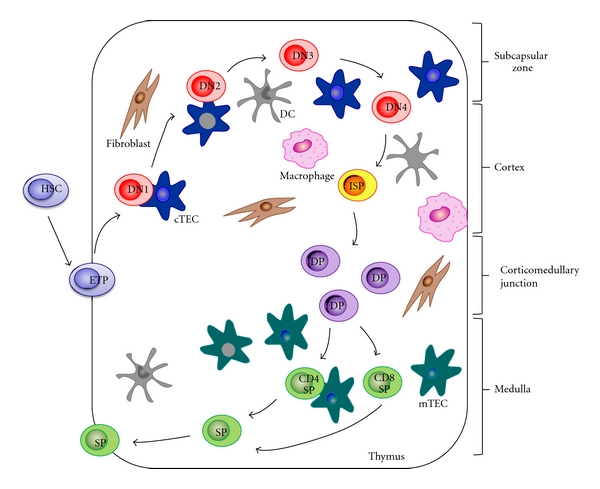
Steps of T-cell development. The lymphoid progenitor cell goes into the thymus through the cortico-medullary junction. DN thymocytes (CD4^−^CD8^−^) migrate across the subcapsular region and then the outer cortex. Interaction between DN cells and cTECs generates DP thymocytes (CD3^+^CD4^+^CD8^+^). Positively selected thymocytes interact with mTECs to complete the maturation process. In the medulla, self-reactive thymocytes are deleted, SP (CD3^+^CD4^+^or CD3^+^CD8^+^) thymocytes are generated, and, eventually, the export of mature T cells from the thymus takes place.

The thymus is the primary lymphoid organ that supports T-cell differentiation and repertoire selection [3, 4]. The intrathymic development of T cells consists of several phases that require a dynamic relocation of developing lymphocytes within multiple architectural structures of this organ. As shown in Figure 1, these steps are (1) the entry of lymphoid progenitor cells into the thymus, (2) the generation of CD4^+^CD8^+^ double positive (DP) thymocytes in the cortex, (3) the positive selection of DP thymocytes in the cortex, and (4) the interaction of positively selected thymocytes with medullary thymic epithelial cells (mTECs) to complete the thymocyte maturation and, eventually, the export of mature T cells from the thymus [5].

Thymus anlagen arises as bilateral structures from the third pharyngeal pouch in the embryonic foregut [6, 7]. The interaction of the epithelial component with the lymphoid progenitor takes place as early as embryonic day 11.5 in mice and at the eighth week of gestation in humans [8, 9].

At an early stage, these precursors have both lymphoid and myeloid potential [10, 11] and are characterized by the expression of the CC-chemokine receptor 9 (CCR9), that, along with the CCR7, plays a central role in this precocious stage of thymus colonization. At this stage of differentiation, lymphoid cells also express the stem- and progenitor-cell markers KIT (also known as CD117), the stem-cell antigen-1 (SCA-1), and the growth-factor-receptor tyrosine kinase type 3 (FLT3) [12–14].

Following the entry into the thymus through the corticomedullary junction, lymphoid progenitor cells begin their commitment toward the T-cell lineage. The developmental pathway is traditionally divided into three subsequent steps, as defined by peculiar immunophenotypic patterns: the CD4^−^CD8^−^ double negative (DN) stage, the CD4^+^CD8^+^ double positive (DP) stage, and the CD4^−^CD8^+^ or CD4^+^CD8^−^ single positive (SP) stage. In mice, an immature single positive (ISP) CD8^+^CD4^−^ cell may be detected between the DN and DP stages. This population can be easily distinguished from the mature SP cell by the high levels of expression of T-cell receptor (TCR) **β** and CD3 and the low level of CD24 (heat stable antigen, HSA). DN cells in mice can be further subdivided based on the expression of CD44 and CD25 in the following populations: CD44^+^CD25^−^(DN1), CD44^+^CD25^+^ (DN2), CD44^−^CD25^+^(DN3), and CD44^−^CD25^−^ (DN4) [15].

From the early T-cell lineage progenitor (ETP) stage to the double-negative 3 (DN3) stage, T-cell differentiation is independent from the TCR and is dependent on the migration through the distinct thymic structures [16]. These phases are regulated by the expression levels of specific transcription factors and by a fine tuned interplay between them (Figure 1).

 At the beginning, ETPs and DN2 cells exhibit a high proliferative capability. Differently, at the DN3 stage, when a fully rearranged TCR occurs, the proliferation stops. In the initial thymocyte development till the DN3 stage, Notch-mediated signals play a pivotal role [17, 18] also supported by signals delivered through the interleukin-7 receptor (IL-7R) [19, 20].

The immature thymocytes journey through the thymus has also the additional effect of promoting the differentiation of thymic stromal precursors into mature thymic epithelial cells, thus playing an important role in the formation of the thymic microenvironment [21–24]. In particular, thymocytes during the DN1-DN3 stages participate to the differentiation process of TEC precursor cells into cortical TECs (cTECs).

**Figure 2:**
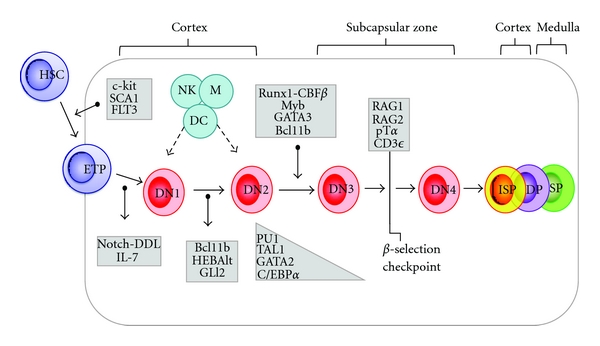
Differential gene expression profile, which modulates the discrete stages of the T-cell development. The lymphoid progenitors, entering into thymus and expressing the markers of HSCs, are primed to Notch and IL-7 signaling until DN1 stage. During the transition DN1/DN2, immature thymocytes lose multilineage potential through the downregulation of genes involved in the differentiation towards other cellular lineages, as PU.1, TAL1, GATA-2, and C/EBP**α**. At the DN2 stage, Myb, GATA-3, HEBalt, GLI-2, and Bcl-11b are upregulated. At the DN3 stage, the genes required for a proper TCR assembly as Rag-1, Rag-2, and pT**α** are expressed, thus leading to the **β**-selection. Following **β**-selection check-point, DN4 cells are fully committed to the TCR**αβ** ^+^ T-cell lineage.

 The DN1 cell thymocytes keep the potential to differentiate into B, T, myeloid, NK, and dendritic cells (DCs) [25–27]. The transition to DN2 is characterized by the upregulation of a number of genes involved in the process, including genes needed for rearrangement and/or expression of the pre-TCR signaling complex components (Figure 2) [28]. At this stage, the thymocytes lose the multilineage potential due to silencing of genes involved in the differentiation towards other cellular lineages. Nevertheless, this potential is not completely lost, since cells with the DN2 phenotype can still differentiate into NK cells, DCs, or macrophages under certain circumstances [29, 30].

 DN2 stage T cells are fully responsive to IL-7 and SCF due to the high expression of IL-7R**α** and c-kit. The DN2 stage is characterized by the upregulation of CD25 molecule (interleukin-2 receptor **α**, IL-2R**α**) and CD90 (Thy-1) [28]. Moreover, the genes which favor the myeloid, NK, and dendritic fate, so-called T-cell antagonists, as PU.1, stem-cell leukemia (SCL also known as TAL1), GATA binding protein-2 (GATA-2), and CCAAT-enhancer binding protein **α** (C/EBP**α**) are silenced before that **β** or **γδ** selection takes place (Figure 2) [31]. During this phase only a few transcription factors, including the zinc-finger transcription factor, the tumor suppressor factor B-cell lymphoma/leukemia 11b (BCL-11b) [32], basic helix-loop-helix (bHLH) transcription factors alternative (HEBalt) [33], and, more transiently, glioma-associated oncogene 2 (GLI-2), a transcription factor involved in the sonic hedgehog signaling [34], are expressed (Figure 2).

The following DN2 to DN3 stage transition requires the expression of different arrays of genes, as Runt-related transcription factor 1-Core binding factor **β** (Runx1-CBF**β**) complexes, the transcription factor Myb, GATA-3, and Bcl-11b, which allow full TCR**β** gene rearrangement in thymocytes, that become competent to undergo **β**-selection [35–37]. Several important events occur during the DN2/3 transition, as the induction of recombinase activating gene-1 (Rag-1) and Rag-2, the upregulation of pre-T**α** (pT**α**), and the rearrangement of TCR**δ** and **γ**. CD3**ε** and IL-7R**α** (CD127) are also upregulated at this phase [38] along with the turn-on of the *lck* tyrosine kinase implicated in the pre-TCR and TCR signaling [39]. At this point, T-cell precursors lose their capability to follow a non-T-cell fate choice [28].

The cells overcoming **β**-selection express the pre-TCR complex on their surface and reach the DN3 stage [40]. Thereafter, the E-proteins E2A and HEB play a crucial role in several processes and are required for the progression of the T-cell development. In fact, these proteins are involved in the TCR gene rearrangement [41], in conferring the competence to undergo **β**-selection, and in the arrest of thymocyte proliferation at the DN3 stage [42].

At the DN3 stage, pre-TCR signaling results in the downregulation of CD25, pT**α**, Rag-1, and Rag-2, which leads to the appearance of DN4 cells. These cells are fully committed to the **αβ** T-cell lineage [43, 44]. After **β**-selection, the thymocytes, which have properly rearranged TCR**β** chains, show a burst of proliferation and a subsequent upregulation of CD8 and then CD4. At this point, the cells become double positive (DP). Eventually, DP cells rearrange TCR**α** gene, leading to TCR**α** assembly into a TCR complex.

The newly generated DP thymocytes are localized in the cortex and express low levels of the TCR**αβ** complex. This DP population consists of T cells with an unselected repertoire [45, 46]. Following that, positive and negative selections take place. In the cortex, the DP thymocytes interact through their TCR with peptide-MHC complexes expressed by stromal cells, as cTECs and dendritic cells [47]. When TCR interacts with low-avidity with the peptide-MHC ligands, DP thymocytes receive survival signals. This process, referred to as positive selection, allows “productive” T cells to potentially react to foreign antigens, but not to self-antigens [5]. Lately, positively selected DP thymocytes are ready to differentiate into SP cells, that is, CD4^+^CD8^−^ or CD4^−^CD8^+^ and relocate into the medulla. At this site, newly generated SP thymocytes are further selected by the medullary stromal cells, including autoimmune regulator- (AIRE-) expressing mTECs. The cells which are reactive to tissue-specific self antigens are deleted, thus avoiding autoimmunity [5]. SP thymocytes egress from the thymus as recent thymic emigrants (RTEs), naïve cells expressing the CD62 ligand (CD62L), also known as lymphocyte- (L-) selectin, CD69, and the CD45RA isoform. These RTE cells are fully mature T cells that exert proper functional capabilities of cell-mediated immunity [48–50].

## 3. Pathogenetic Mechanisms of T-Cell Defects

Most of the pathogenic mechanisms underlying primary T-cell disorders are related to molecular alterations of genes selectively expressed in hematopoietic cells. However, since the differentiation process requires a crosstalk among thymocytes and thymic microenvironment, a severe T-cell defect may also be due to alteration of the stromal component of the thymus.

T-cell disorders include a wide spectrum of disorders that affect T-cell development and/or function. The severity of the T-cell defect varies a lot ranging from the syndrome of severe combined immunodeficiency (SCID), characterized by a complete absence of T-cell functions to combined immunodeficiency disorders, in which there are a low number of T cells whose function is not adequate [51].

SCIDs comprise a heterogeneous group of monogenic disorders characterized by a virtual lack of functional peripheral T cells. To date, more than 20 different genetic defects involved in the pathogenesis of SCID in humans have been identified [52, 53]. Typically, patients with SCID show a severe defect in T-cell differentiation and a direct or indirect impairment of B-cell development and function. On the basis of the involvement of different cell lines in the pathogenesis of the disease and of the subsequent different clinical phenotypes, SCIDs have been till now classified according to the presence or absence of T, B, and NK cells (Table 1). Impaired survival of lymphocyte precursors is observed in reticular dysgenesis (RD) and in adenosine deaminase (ADA) deficiency. In RD the mutations of the adenylate kinase 2 gene (AK2) result in increased apoptosis of myeloid and lymphoid precursors. As a consequence, patients with RD show marked lymphopenia and neutropenia [54, 55]. ADA deficiency is characterized by the accumulation of high intracellular levels of toxic phosphorylated metabolites of adenosine and deoxyadenosine that cause apoptosis of lymphoid precursors in the bone marrow and thymus [56, 57].

The majority of SCIDs in human subjects derive from alterations of the cytokine-mediated signaling apparatus. SCID-X1 represents the most common form of SCID and is caused by mutations of the IL-2 receptor **γ** gene (IL-2R**γ**), which encodes for the common **γ**-chain (**γ**-c) shared by cytokine receptors, including those for IL-2, IL-4, IL-7, IL-9, IL-15, and IL-21. Patients usually have few or no T and NK cells but a normal or elevated number of B cells which fail to produce immunoglobulins normally [58]. **γ**-c also plays effects on cell cycle control and participates to the growth of tumoral cells, as well [59, 60]. Defects of JAK3, an intracellular tyrosine kinase physically and functionally coupled to **γ**-c, result in a syndrome whose immunologic phenotype is undistinguishable from that of SCID-X1 [61]. Mutations in the gene encoding for the **α**-chain of the IL-7R abrogate T lymphocyte development but leave B and NK cell development intact [62]. Mutations in critical genes needed for the expression of pre-T-cell receptor, as Rag-1 and Rag-2, result in a functional inability to form antigen receptors through genetic recombination, compromising the production of functional T cells. These proteins recognize recombination signal sequences and introduce a DNA double-stranded break, permitting V, D, and J gene rearrangements [63, 64]. Lymphocyte phenotype differs from those of patients with SCID caused by **γ**-c, Janus kinase-3 (Jak-3), IL-7R**α**, or ADA deficiencies in that they lack both B and T lymphocytes since pre-TCR and pre-B-cell receptor (BCR) share similar molecular mechanisms requiring Rag-1 and 2 expression [65]. Defects of pre-TCR and pre-BCR expression might also reflect mutations in genes that encode proteins involved in nonhomologous end-joining (NHEJ) and DNA repair and, in particular, Artemis, DNA protein-kinase catalytic subunit (DNA-PKcs), Cernunnos/XLF, and DNA ligase IV [65–69]. In all these diseases, the generation of both T and B lymphocytes is severely compromised. However, it should be noted that a functional T-cell defect may also be due to infections [70, 71] or during the reconstitution phase following stem cell transplantation [72].

It is noteworthy that all the genes whose alterations lead to the above mentioned forms of SCID selectively impair the lymphocyte functionality and the ability of these cells to proceed in the developmental pathway. In some cases, as in the case of TrkA mutation [73], the gene has pleiotropic effects resulting in complex multisystemic disorders associated to immunodeficiency.

## 4. The Murine Model of Athymia: nu/nu Mice

The first example of SCID not primarily related to a hematopoietic cell abnormality but rather to an intrinsic thymic epithelial cell defect is the Nude/SCID phenotype, whose identification contributed to unravel important issues of T-cell ontogeny.

The “nude” phenotype, identified for the first time in mice, results from inactivating mutations in a single gene, originally named winged-helix-nude (whn) and recently known as forkhead box n1 (foxn1) [74]. This murine model was described by Flanagan in 1966, when spontaneously appeared in the Virus Laboratory of Ruchill Hospital in Glasgow (UK) [75–77]. Mice homozygous for the mutation “nude” are hairless, have retarded growth, decreased fertility, and die by 5 months of life for infections. The hairlessness is due to the coiling of the incomplete hair shafts in the dermis caused by the absence of free sulfhydryl groups in the midfollicle region [78]. The “nude” *foxn1* gene does not affect the growth of hair follicles, but the epidermal differentiation process, regulating the balance between proliferation and differentiation of keratinocytes in the hair follicle [79, 80]. The “nude” mice are affected by severe infertility and show small ovaries with low egg counts in the females and no motile sperm in the males [78]. This condition may be the result of changes in hormonal status, as demonstrated by altered serum levels of estradiol, progesterone, and thyroxine [81]. The thymus is absent at birth [82] and there are very few lymphocytes in the thymus dependent areas of the spleen and lymph nodes [83].

Since the abnormal, or even absent, thymus is the hallmark of the “nude” phenotype, these animals develop a profound T-cell deficiency and a severely impaired immune response of either cell-mediated and, indirectly, humoral immunity. In “nude” mice, when the thymus is present in the first days of life, it reveals no normal structure, consisting of a thymic rudiment composed of vesicles or canaliculi delimited by epithelial-like cells, with no trace of lymphoid cells. By the day 14, the “nude” thymus is much smaller compared to the normal [84].

Nu/nu mice show lymphopenia and also low immunoglobulin levels. In the absence of normal T cells originated from the thymus, the development of the antibody forming cells is delayed, although “nude” mice do not lack precursors of antibody forming cells. This indicated that antibody forming cells may mature in the absence of the thymus, albeit at a slower rate [85]. In “nude” mice lymph nodes, the outer cortex with primary nodules and the medullary cords are normal. In the spleen sections from the nu/nu mice, the proportion of red to white pulp is greater than normal and, in some cases, an unusually high number of megakaryocytes are seen in the red pulp. In some spleens, Malpighian follicles, although present, are fewer and smaller than in controls and a depletion of lymphocytes is constant in the close proximity of the central arteriole in the thymus-dependent area. The depletion in the splenic thymus-dependent areas is not as prominent as in the lymph nodes [83]. In man, the prototype of an athymic disorder has long been considered the DiGeorge's Syndrome (DGS), even though main features of athymic murine model and human disease, including immunological signs, are not completely overlapping.

## 5. The Athymic DiGeorge Syndrome

The DGS, along with velocardiofacial syndrome and conotruncal anomaly face syndrome, is frequently associated to a common heterozygous intrachromosomal deletion in 22q11.2. However, a DGS-like phenotype can have alternative etiologies, including maternal diabetes, fetal alcohol syndrome, and teratogenesis, even though the molecular mechanisms underlying these forms are still unknown [86]. DGS has an estimated incidence of 1 in 4000 live births [87, 88] and, thus, it is the most common microdeletion syndrome in humans and the second most common chromosomal disorder after Down's syndrome. The deletion is due to a meiotic nonallelic homologous recombination between flanking 250 kilobases (kb), mapping in 22q11.2 chromosomal region and consisting in low-copy repeats/segmental duplications in the termed LCR22 [89, 90]. Although most cases of DGS occur as *de novo* deletions, approximately 5% of cases are inherited as an autosomal dominant trait [91–93]. In the 90% of patients, a hemizygous 3 Mb deletion, containing about 30 genes [89, 90, 94, 95], is found, whereas approximately 8% of patients carry a smaller deletion of 1.5 Mb, encompassing 24 genes [96], even though no difference in the clinical presentation is appreciable in the smaller deletion [86].

 The main features of this syndrome are mild facial dysmorphism, submucous cleft palate, velopharyngeal insufficiency, speech delay, recurrent infections, variable immunodeficiency secondary to thymic aplasia or hypoplasia, and cardiac anomalies [97, 98]. Most of the patients have learning disabilities and behavioral disorders, including schizophrenia in some cases [99–102]. Children with the DGS, according to the aplasia or hypoplasia of the thymus, are classified as complete or partial DGS. The “complete” form represents a small percentage of patients, accounting to the 0.5% of all patients. These patients show a severe combined immunodeficiency phenotype with near absent T lymphocytes. The majority of patients have a “partial” phenotype and an immune defect usually manifesting as mild to moderate T lymphocytopenia. The T-cell proliferation is usually normal or in very few cases low normal. These patients have been reported to have a moderate increase of the number of infections than predicted on the basis of the immunological impairment, suggesting that anatomical defects, gastroesophageal reflux, allergies, cardiac disease, and poor nutrition may also contribute to recurrent infections [103]. It should be underlined that never “partial” DGS patients have severe infections as reported in SCID and, moreover, T-cell proliferation is usually normal. A moderate CD4 lymphocytopenia with low to normal CD8 T lymphocytes is usually found. An age-related decrease of T lymphocytes is also seen in DGS patients. TCR repertoire analysis in 22q11.2 deletion patients has shown significant oligoclonal peaks and V**β** family dropouts when compared to controls. In a study of nine patients with a negative infectious history, a decreased diversity in CD4^+^ and CD8^+^ TCR repertoire, using both flow cytometric and third complementarity determining region (CDR3 spectratyping) fragment analysis, has been documented [104]. In another study, the spectratyping showed alterations in the repertoire, which, however, improved over the time [105].

Immune deficiency in these patients seems to be associated to an increased incidence of autoimmune diseases [106–108], in particular cytopenias [109, 110], arthritis [111], and endocrinopathies [112].

The chromosomal region usually deleted contains several genes, which may be candidate of the DGS phenotype. TBX1, which belongs to the family of T-box transcription factors, which share a common DNA binding domain is called “T-box” [113]. A specific role for Tbx1 in DGS and thymus development came out from the peculiar expression pattern in both the third pharyngeal pouch endoderm and the adjacent mesenchyme and not in the neural crest cells [114]. Furthermore, the homozygous loss of *Tbx1* causes thymic hypoplasia, as well [96, 115–117]. Of note, mice heterozygous for a null allele of *Tbx1* demonstrate only a mild phenotype without thymus anomalies [118]. Thus, evidence would suggest, at least in mice, that gene dosage of *Tbx1* is crucial in the pathogenesis of DGS. However, in the same region there are other genes potentially implicated in the pathogenesis of DGS, such as *Crkl*, which encodes an adaptor protein implicated in growth factor and adhesion molecule signaling. Homozygous *Crkl* gene deletion results in multiple defects in neural crest derivatives including aortic arch arteries, thymus, and craniofacial structures [96] and in prenatal death. However, the deletion at the heterozygous state does not cause any clinical sign, thus indicating that a combination of gene alterations is needed for the full expressivity of the phenotype [119].

## 6. The Human Nude/SCID Phenotype

The human equivalent of the “nude” murine phenotype was first described in two sisters in 1996, after more than 30 years from the initial mouse description and, subsequently, associated to *FOXN1* gene alterations.

**Figure 3:**
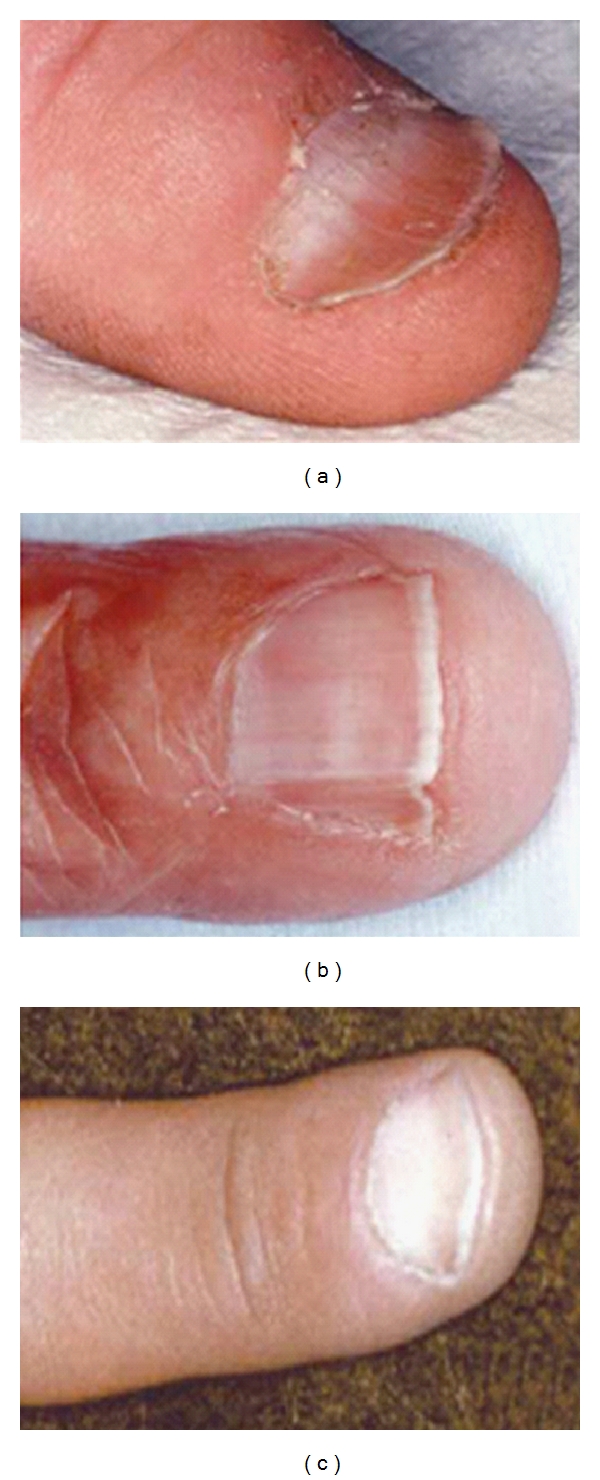
Nail dystrophy patterns in subjects carrying heterozygous mutations in FOXN1 gene: (a) koilonychias, (b) canaliform dystrophy, and (c) leukonychia.

The human Nude/SCID is an autosomal recessive disorder [120], whose hallmark is the T-cell immunodeficiency due to the complete absence of the thymus. This immunodeficiency presents in a quite similar fashion to the classical SCID phenotype, thus being more severe than DGS. Along with the severe infections, other features of the syndrome are ectodermal abnormalities, as alopecia and nail dystrophy [121]. Of note, the nail dystrophy can be observed also in subjects carrying the genetic alteration in heterozygosity. The most frequent nail alteration is the koilonychia (spoon nail), characterized by a concave surface and raised edges of the nail plate, associated with significant thinning of the plate itself; a canaliform dystrophy associated to a transverse groove of the nail plate (Beau line) may also be found (Figure 3). However, the most specific phenotypic alteration is leukonychia, characterized by a typical arciform pattern resembled to a half-moon and involving the proximal part of the nail plate. These alterations of digits and nails have also been reported in a few strains of “nude” mice. FOXN1 is known to be selectively expressed in the nail matrix where the nail plate originates, thus confirming that this transcription factor is involved in the maturation process of nails and suggesting nail dystrophy as an indicative sign of heterozygosity for this molecular alteration [121].

Interestingly, additional studies have also reported on anomalies of brain structures, suggesting a potential role of this transcription factor in brain embryogenesis, as also suggested by its expression in epithelial cells of the developing choroids plexus, a structure filling the lateral, third, and fourth ventricles. However, the severe neural tube defects, including anencephaly and spina bifida, have been only inconstantly reported, thus probably indicating that the genetic alteration represents a cofactor and is not sufficient *per se* to alter brain embryogenesis. The anomalies of brain structure have been considered potentially responsible for the high rate of mortality *in utero* observed in the geographic area with the high frequency of *FOXN1* alteration [122].

Prenatal alteration of the *FOXN1* gene in humans prevents the development of the T-cell compartment as early as at 16 weeks of gestation [123]. By contrast, stem cells, B, and NK lymphocytes are normal. CD4^+^ cells are more affected than CD8^+^ cells, even though the latter are also profoundly reduced. No CD4^+^CD45RA^+^ naive cells can be usually found [123]. CD8 cells coexpressing CD3 are very scarce and a few CD3^+^CD8^+^CD45RA^+^ naïve cells can be detected [123]. Overall, a substantial reduction of T cells bearing TCR**αβ**, but not of lymphocytes expressing TCR**γδ**, is observed [123]. TCR gene rearrangement, although altered, occurs to some extent, suggesting the possibility of an extrathymic and FOXN1-independent site of differentiation. However, it should be emphasized that these few T cells, which escape the blockage, are unable to sustain a productive immune response into the periphery.

Taken together, the data so far available underline the crucial role of FOXN1 in the early prenatal stages of T-cell ontogeny in humans [123].

## 7. Role of FOXN1 in Immune System

*FOXN1 *belongs to the forkhead-box gene family that comprises a diverse group of “winged helix” transcription factors implicated in a variety of cellular processes: development, metabolism, cancer, and aging [124]. These transcription factors share the common property of being developmentally regulated and of directing tissue specific transcription and cell fate decisions. While during embryogenesis *FOXN1* is expressed in several mesenchymal and epithelial cells, including those of the liver, lung, intestine, kidney, and urinary tract, later, its expression is confined to skin and thymus epithelia, where FOXN1 is absolutely required for the normal differentiation of hair follicles and TECs.

*FOXN1* gene, spanning about 30 kb [125, 126], is an epithelial cell-autonomous gene and is highly conserved in sequence and function in rodents and humans. Interestingly, an extensive screening of cDNA clones obtained from skin cells revealed the presence of two different noncoding first exons [126], the exons 1a and 1b, that undergo to alternative splicing to either of two splice acceptor sites of the exon 2, located upstream of the initiation codon. This suggests the presence of two distinct promoters of exons 1a and 1b [125]. The alternative usage of the exon 1a or 1b seems to direct the tissue specificity [126], in that promoter 1a is active in thymus and skin, while promoter 1b is active only in skin.

The molecular mechanisms by which *FOXN1* expression and activity are regulated are only incompletely understood. It is suggested that FOXN1 might, subsequently, upregulate the expression of fibroblast growth factor (FGF) receptors, which in turn modulate the thymic stroma differentiation and thymopoiesis [127]. *In vitro* exposure of thymic epithelial cells to some Wnt proteins is sufficient to upregulate FOXN1 protein expression in both an endocrine and paracrine fashion [128]. Wnts belong to a large family of secreted glycoproteins that have important roles in cell-fate specification [127].

The prenatal thymus development, the maintenance of a proper thymic microenvironment, and the efficient T-cell production require an appropriate crass-talk between thymocytes and thymic stromal cells [12]. Postnatally, the thymic involution results in dramatically reduced T-cell generation in an age-dependent fashion [129].

Indeed, recent evidence has implicated both TEC- and hematopoietic stem cell- (HSC-) intrinsic defects in involution of the organ [130–133]. *Foxn1* is expressed in all TECs during initial thymus organogenesis and is required for the initial phase of their differentiation [75, 134, 135]. *Foxn1* exerts an important role [136] in inducing both cortical and medullary differentiation [137, 138]. Although foxn1 has long been studied, most of the studies thus far available are restricted to fetal differentiation process, while its postnatal role in the mature thymus still remains to be fully elucidated.

 However, it is largely unknown whether the role of foxn1 in the thymus and skin is identical. One important difference is that foxn1 is involved in morphogenesis of the three-dimensional thymic microstructure, which is important for the functionality of the thymus [139]. Moreover, the differentiation of the immature epithelial cells into functional cTECs and mTECs is foxn1-dependent. In particular, foxn1 mainly regulates TEC patterning in the fetal stage [140] and TEC homeostasis in the postnatal thymus [141]. TECs are implicated in either thymus organogenesis or in most stages of maturation of thymocytes [142, 143]. The inborn null mutation in *foxn1* [76] causes a differentiation failure in TECs thereby halting thymic development at a rudimentary stage. The thymic lobar architecture is still present but the epithelial cells lack the ability to induce the entrance of hematopoietic precursor cells (HPCs) into the epithelial cluster and thus preclude the generation of thymocytes [144]. These results argue strongly for a failure in thymocytes-epithelial crosstalk, thus, explaining the blockage of thymic lymphopoiesis [75, 136]. The organ is, therefore, an alymphoid two-dimensional (2D) rudiment with a cystic structure [72, 82, 120, 123].

Because of the significant expression levels of FOXN1 in skin elements, keratinocytes have been successfully used to support a full process of human T-cell development in vitro, resulting in the generation of mature T cells from HPCs. This finding would imply a role for skin as a primary lymphoid organ [145].

## 8. Conclusion and Future Research

Primary T-cell defects are rare disorders, accounting for approximately 11% of reported PIDs. These disorders include a wide spectrum of diseases that affect T-cell development and/or function. The pathogenic mechanisms are mostly related to molecular alterations of genes selectively expressed in hematopoietic cells. However, they can also be due to alterations of the stromal component of the thymus, which is the primary lymphoid organ that supports T-cell differentiation and repertoire selection. In this organ, the dynamic relocation in multiple architectural structures requires the cross-talk between thymocytes and thymic microenvironment. The Nude/SCID syndrome results from inactivating mutations in the gene encoding the *FOXN1* transcriptional factor selectively expressed in skin and thymic epithelia. In mice and humans its alteration leads to thymic agenesia and severe T-cell deficiency. The Nude/SCID immunodeficiency is much more severe than DGS, indicating that the *FOXN1* expression is absolutely required for an efficient production of mature T cells. The studies on the human Nude/SCID phenotype greatly contributed to unravel important issues of the T-cell ontogeny and, in the near future, may help define potential extrathymic and thymus-independent sites of differentiation in man.

## Figures and Tables

**Table 1:** SCIDs classification. SCIDs have been so far classified according to the presence or absence of T, B, and NK cells, as a consequence of different molecular defects.

Lymphocyte phenotype	Gene defect	Form of SCID
T^−^B^−^NK^−^	Adenylate kinase	Reticular dysgenesis
	Adenosine deaminase	ADA deficiency
T^−^B^+^NK^−^	IL-2R*γ*	SCID-X1
	Jak3	SCID-AR
T^−^B^+^NK^+^	IL-7R*α*	IL-7R*α* deficiency
T^−^B^−^NK^+^	Rag-1 or Rag-2 artemis	Omenn syndrome Artemis deficiency
